# Optimization of the Extraction Process and Biological Activities of Triterpenoids of *Schisandra sphenanthera* from Different Medicinal Parts and Growth Stages

**DOI:** 10.3390/molecules29102199

**Published:** 2024-05-08

**Authors:** Qiaozhu Zhao, Jianhua Li, Qianqian Shang, Jiafang Jiang, Han Pu, Xilin Fang, Xiaolu Qin, Jia Zhou, Nongxue Wang, Xiaorui Wang, Wei Gu

**Affiliations:** National Engineering Laboratory for Resource Development of Endangered Crude Drugs in Northwest China, The Key Laboratory of Medicinal Resources and Natural Pharmaceutical Chemistry, The Ministry of Education, College of Life Sciences, Shaanxi Normal University, Xi’an 710119, China; qiaozhuzhao@snnu.edu.cn (Q.Z.); lijianhua@snnu.edu.cn (J.L.); shangqianqian@snnu.edu.cn (Q.S.); j2744895462@suun.edu.cn (J.J.); p2272486525@snnu.edu.cn (H.P.); fangxilin@snnu.edu.cn (X.F.); qinxlu@snnu.edu.cn (X.Q.); zhoujia666@snnu.edu.cn (J.Z.); wangnongxue@snnu.edu.cn (N.W.)

**Keywords:** *Schisandra sphenanthera*, total triterpenoids, different medicinal parts, growth stages, antioxidant, antibacterial

## Abstract

*Schisandra sphenanthera* Rehd. et Wils., as a traditional Chinese medicine, has important medicinal value. In the market, the availability of the fruit of *S. sphenanthera* mainly relies on wild picking, but many canes and leaves are discarded during wild collection, resulting in a waste of resources. The canes and leaves of *S. sphenanthera* contain various bioactive ingredients and can be used as spice, tea, and medicine and so present great utilization opportunities. Therefore, it is helpful to explore the effective components and biological activities of the canes and leaves to utilize *S. sphenanthera* fully. In this study, the response surface method with ultrasound was used to extract the total triterpenoids from the canes and leaves of *S. sphenanthera* at different stages. The content of total triterpenoids in the leaves at different stages was higher than that in the canes. The total triterpenoids in the canes and leaves had strong antioxidant and antibacterial abilities. At the same time, the antibacterial activity of the total triterpenoids against *Bacillus subtilis* and *Pseudomonas aeruginosa* was stronger than that against *Staphylococcus aureus* and *Escherichia coli*. This study provides the foundation for the development and utilization of the canes and leaves that would relieve the shortage of fruit resources of *S. sphenanthera*.

## 1. Introduction

*Schisandra sphenanthera* Rehd. et Wils., a deciduous vine [1,2], is mainly distributed in the central, western, and southwestern regions of China, especially in the Qinling Mountains [3,4]. As a Traditional Chinese Medicine (TCM), *S. sphenanthera* is mainly used to treat coughs and asthma, night sweats, palpitations and insomnia, enuresis, and so on [2]. It has also shown significant bioactivities, such as antioxidant, immunomodulatory, and antimicrobial activities [5]. The medicinally active components of *S. sphenanthera* include lignans, essential oils, polysaccharides, triterpenoids, organic acids, and so on [5,6,7,8]. It was reported that lignans, essential oils, and polysaccharides from *S. sphenanthera* may be responsible for its bioactivity with a variety of beneficial effects, including antioxidant, immunomodulatory, hepatoprotection, and antibacterial activities [8,9,10]. Therefore, there have been many studies focusing on the polysaccharides, lignans, and essential oils of *S. sphenanthera*, while current research on triterpenoids mainly focuses on the separation and identification of single components [11,12,13]. It has been found that the triterpenoids of *S. sphenanthera* can promote the growth of endophytic bacteria and fungi, thereby promoting the growth of *S. sphenanthera* and the production of secondary metabolites [14].

Triterpenoids are a class of plant metabolites synthesized from the common intermediate 2,3-oxidized squalene, whose cyclization and rearrangement lead to the formation of triterpenes (with pentacyclic scaffolds), while further enzymatic modifications lead to the occurrence of more than 20,000 different structures in nature [15]. Due to the diversity of their structure, triterpenoids exert a wide range of biological activities, including anti-inflammatory, antibacterial, hepatoprotective, and anticancer properties, and have important pharmaceutical and industrial applications [16,17]. The triterpenoids in the Schisandraceae family mainly include various cycloalkane-type triterpenoids, lanosterane-type triterpenoids, and schinortriterpenoids [18,19,20]. Schinortriterpenoids are the characteristic components that only occur in the Schisandraceae family. In contrast to other triterpenoids, schinortriterpenoids exhibit the features of 3,4-oxidative cleavage, 9,10-cleavage with ring extension, decarboxylation of C-18 or C-28, and five- or six-membered lactone rings on the side chain [18]. The structural diversity of triterpenoids leads to a variety of biological activities, such as hepatoprotective, neuroprotective, antiviral, and anti-inflammatory activities, especially in the case of schinortriterpenoids [18,19].

Extraction methods for triterpenoids include traditional extraction methods and new extraction technologies. The traditional extraction methods have the disadvantages of a long drying time and low efficiency, such as reflux extraction and Soxhlet extraction [21,22]. Therefore, various new extraction technologies are increasingly being used to extract the natural products from TCMs, including supercritical fluid extraction [22] and ultrasonic-assisted extraction [23]. Among these extraction technologies, ultrasonic-assisted extraction is widely used for the extraction of triterpenoids due to its simplicity, rapidity, and high efficiency [23]. The separation and purification techniques for triterpenoids mainly include column chromatography on silica gel [24], thin-layer chromatography [25], high-speed countercurrent chromatography [26], macroporous resin adsorber chromatography [27], and recrystallization [28]. Macroporous resin adsorber chromatography is often used for the separation and purification of triterpenoids and has the advantages of reusability, low price, good adsorption efficiency, and fast exchange rate [29,30].

The most important medicinal constituent of *S. sphenanthera* is the fruit, which is mainly obtained by picking in the wild. In addition, the active ingredients in the canes and leaves are similar to those of the fruits and are also used to treat abdominal pain, gastroenteritis, falls, rheumatic joint pain, and so on [31]. It is a common phenomenon to cut off the vine to get the fruits, which destroys the resources of *S. sphenanthera*. The canes and leaves that are cut off every year in the wild cultivation areas are usually thrown away and not used. So far, there have been few studies at home or abroad on the extraction technology and biological activities of total triterpenoids from the canes and leaves of *S. sphenanthera*, and there are also few studies on the distribution of total triterpenoids in different parts of the plant and the best harvesting time. Therefore, it is worthwhile to investigate the extraction, antioxidation, and bacteriostasis of triterpenoids from the canes and leaves of *S. sphenanthera*.

In this study, the canes and leaves of *S. sphenanthera* at different stages were used as materials. The research contents of this study were addressed as follows. (a) Based on single-factor experiments, the total triterpenoids from the canes and leaves of *S. sphenanthera* were determined using response surface methodology. (b) The distribution and dynamic changes in the triterpenoids in the canes and leaves of *S. sphenanthera* at different growth stages were investigated, and the optimal utilization ratio and harvest time were determined. (c) The antioxidant and antibacterial abilities of the total triterpenoids in *S. sphenanthera* canes and leaves after purification were investigated to analyze the dynamic changes in the biological ability of the total triterpenoids. It is expected that this study will provide data to support the further development of diversified deep processing of *S. sphenanthera* and relieve the pressure on the fruiting resources of *S. sphenanthera*.

## 2. Results

### 2.1. Optimization of Extraction Rate

#### 2.1.1. Influence of Single Factors on the Extraction Rate

**Figure 1:**
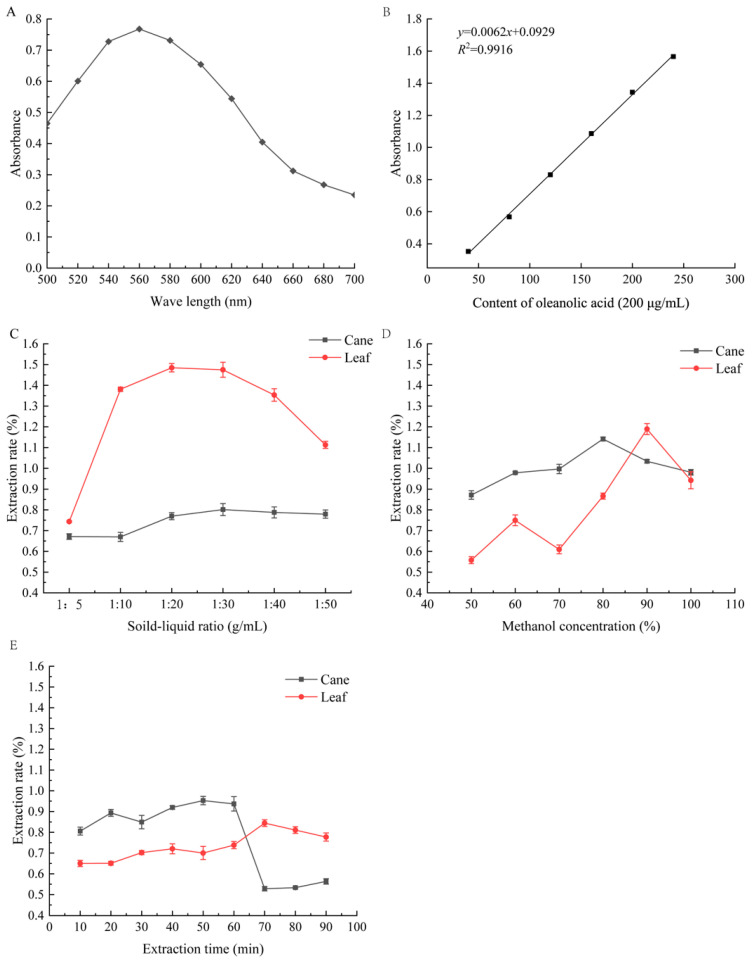
The effects of maximum absorption peak (**A**) and standard curve (**B**) of oleanolic acid and single factor experiment on the extraction rate of triterpenoids from the canes and leaves of *S. sphenanthera* (**C**–**E**). (**C**) Solid–liquid ratio; (**D**) methanol concentration; (**E**) extraction time.

In this experiment, the influence of three single factors was judged according to the extraction rate of total triterpenoids, which was calculated according to the standards of oleanolic acid (Figure 1). With the increase in the solid–liquid ratio from 1:10 to 1:30 g/mL, methanol concentration from 50% to 80%, and extraction time from 30 to 60 min, the extraction rate of the total triterpenoids in the canes gradually increased. However, the extraction rate of total triterpenoids in the leaves increased sharply with the increase in the solid–liquid ratio from 1:5 to 1:20 g/mL, methanol concentration from 70% to 90%, and extraction time from 50 to 70 min (Figure 1C–E). Therefore, considering the extraction volume, solvent consumption, and production cost, the solid–liquid ratio of 1:20~1:40 g/mL, methanol concentration of 75~85%, and extraction time of 40~60 min were selected to further optimize the extraction of total triterpenoids from canes. The extraction of total triterpenoids from leaves was optimized by selecting the solid–liquid ratio as 1:10~1:30 g/mL, methanol concentration as 85~95%, and extraction time as 60~80 min (Tables S1 and S2).

#### 2.1.2. Optimization of Extraction for Total Triterpenoids by RSM

**Figure 2:**
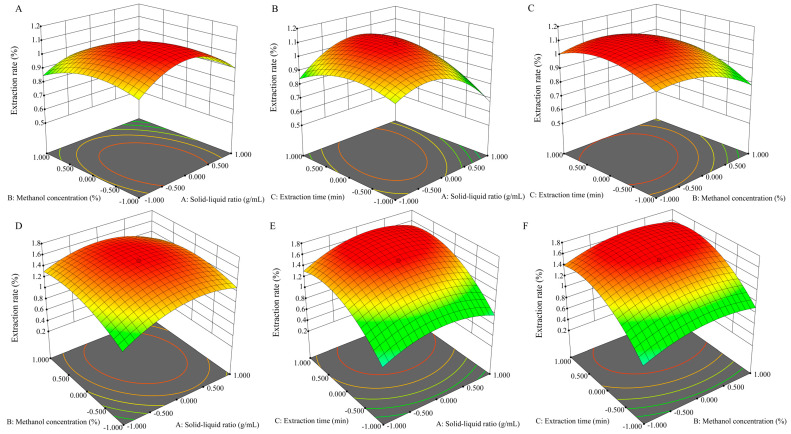
The 3D response surface diagram of the interaction of each factor on the extraction rate of triterpenoids from the canes and leaves of *S. sphenanthera*. (**A**) Solid–liquid ratio and methanol concentration of canes; (**B**) solid–liquid ratio and extraction time of canes; (**C**) methanol concentration and extraction time of canes; (**D**) solid–liquid ratio and methanol concentration of leaves; (**E**) solid–liquid ratio and extraction time of leaves; (**F**) methanol concentration and extraction time of leaves.

Based on the single-factor experiment, the RSM method was constructed by central combination design (CCD) of Design-Expert v8.0.6.1 software to optimize the process conditions (Figure 2, Tables S1 and S2). According to the fitting analysis of the experimental results in Tables S3 and S4, a model of the relationship between the total triterpenoid extraction rate (Y) of the canes and leaves of *S. sphenanthera* and the independent variables was established with Equations (1) and (2). Regression analysis showed that the extraction of the total triterpenoid was predicted by the second-order equation.

(1) $$Y_{1}=1.110-0.038A_{1}-0.065B_{1}+0.045C_{1}-0.030A_{1}B_{1}+0.082A_{1}C_{1}+0.029B_{1}C_{1}-0.170{A_{1}}^{2}-0.085{B_{1}}^{2}-0.096{C_{1}}^{2}$$

(2) $$Y_{2}=1.640+0.069A_{2}+0.089B_{2}+0.340C_{2}-0.080A_{2}B_{2}+0.033A_{2}C_{2}-0.005B_{2}C_{2}-0.240{A_{2}}^{2}-0.170{B_{2}}^{2}-0.310{C_{2}}^{2}$$

where *A*_1_ and *A*_2_ are the solid–liquid ratio (g/mL), *B*_1_ and *B*_2_ are the methanol concentration (%), *C*_1_ and *C*_2_ are the extraction time (min), *Y*_1_ and *Y*_2_ are the extraction rate of total triterpenoids (%).

According to the variance analysis of the model of total triterpenoids from canes and leaves (Tables S3 and S4), the significance test of the regression equation (*p* < 0.001) showed that the quadratic multiple regression model was extremely significant, and the *p*-values were 0.098 (>0.050) and 0.070 (>0.050), respectively, indicating that the model fitted well with the experiment and the experimental error was small. The independent variables (*A*, *B*, *C*) and the three quadratic terms (*A*^2^, *B*^2^, *C*^2^) had significant effects on the extraction rate of total triterpenoids (*p* < 0.05), and the solid–liquid ratio also had significant interactions with the extraction time (*A*_1_*C*_1_) (cane) and the solid–liquid ratio with methanol concentration (*A*_2_*B*_2_) (leaf) (*p* < 0.01). The coefficients of variation (CV) of 3.940% and 4.400% showed that the model had credibility, and could be used to analyze and predict the extraction rate of total triterpenoids from the canes of *S. sphenanthera*.

The order of influence of the various factors on the extraction rate of the total triterpenoids from canes and leaves were as follows: methanol concentration > extraction time > solid–liquid ratio, and extraction time > methanol concentration > solid–liquid ratio, respectively (Figure 2, Tables S3 and S4). In this study, with the opening of the 3D response surface downward, the response value of the total triterpenoid will also increase with the increase in factor values (*AB*, *AC*, *BC*), and the response value will gradually decrease with the increase in factor values after increasing to the extreme value (Figure 2). The software predicted that the optimum extraction conditions for total triterpenoids from canes were as follows: solid–liquid ratio 1:29.580 g/mL, methanol concentration 78.250%, extraction time 51.630 min, and the maximum extraction rate was 1.130%. For the convenience of operation, we adopted the conditions of a solid–liquid ratio of 1:30 g/mL, methanol content of 78%, and extraction time of 52 min. The actual extraction rate was 1.14 ± 0.23%, which was close to the expected value. Similarly, the optimum extraction conditions for total triterpenoids from leaves predicted by the software were as follows: the ratio of solid to liquid was 1:21.450 g/mL, the concentration of methanol was 91.090%, the extraction time was 75.540 min, and the maximum extraction rate was 1.745%. When the ratio of solid to liquid was 1:21 g/mL, the concentration of methanol was 91% and the extraction time was 76 min, the actual extraction rate was 1.78 ± 0.17%, which was close to the expected value.

### 2.2. Total Triterpene Content and Purification

**Figure 3:**
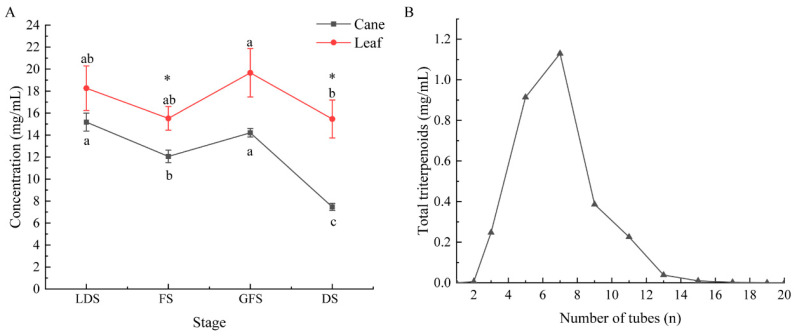
Concentrations of total triterpenoids in canes and leaves of *S. sphenanthera* at different stages (**A**) and analytic curve of D-101 resin (**B**). LDS: leaf development stage; FS: flowering stage; GFS: green fruit stage; DS: defoliate stage. Different lowercase letters indicate that the content of total triterpenoids varied significantly in the same parts at different stages (*p* < 0.05). ‘*’ indicates that the difference of total triterpenoids in different parts in the same stage was significant (*p* < 0.05).

The total triterpenoids were extracted from different parts of *S. sphenanthera* plants harvested at different growth stages by the optimized extraction technology. The content of total triterpenoids in the different parts showed dynamic changes during the growth process, showing a trend of first decreasing, then increasing, and then decreasing (Figure 3A). The total triterpenoid content of the leaves (15.47~19.66 mg/g) was always higher than that of the canes (7.47~15.18 mg/g). The content of total triterpenoids in the leaves during the green fruit stage (GFS) was the highest (19.66 mg/g), and the content during the defoliate stage (DS) was the lowest (15.47 mg/g). The content decreased from the GFS to the DS with a significant difference (p < 0.05), and the other changes were not significant. The content of total triterpenoids in the canes was significantly different during the growth process (*p* < 0.05), with the highest content in the leaf development stage (LDS) (15.18 mg/g), and the lowest content in the DS (7.47 mg/g). There was a significant difference in the total triterpenoid content between the canes and leaves at the flowering stage (FS) and the DS (*p* < 0.05). 

The triterpenoids purified by MRAC gave a good desorption effect, and only one peak appeared (Figure 3B), indicating that the elution was relatively concentrated. The purity of the purified total triterpenoids was 75.02 ± 3.83%, which was about 2.8-fold higher than before. The average recovery rate of purification was 26.45 ± 3.75%. The purification effects on the canes and leaves of *S. sphenanthera* in different stages are shown in Table S5.

### 2.3. Antioxidant Activity Determination

**Figure 4:**
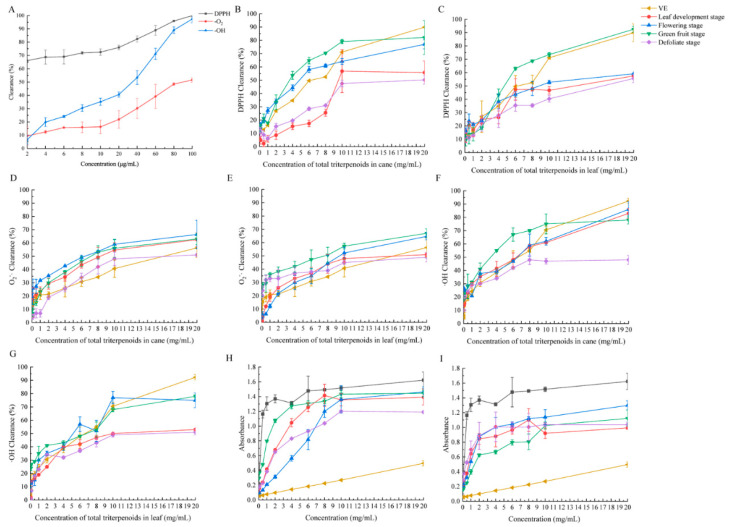
The antioxidant capacity of total triterpenoids of *S. sphenanthera*. (**A**) VC clearance capacity of DPPH, O_2_^−^·, and ·OH; (**B**,**D**,**F**) cane clearance capacity of DPPH, O_2_^−^·, and ·OH; (**C**,**E**,**G**): leaf clearance capability of DPPH, O_2_^−^·, and ·OH; (**H**) reduction ability of canes; (**I**) reduction ability of leaves. The black line represents VC in (**H**,**I**).

The total triterpenoids in the canes and leaves of *S. sphenanthera* for DPPH, O_2_^−^·, ·OH radicals have a strong scavenging effect, and there is a dose–response relationship between the concentration and scavenging rate (Figure 4 and Table S6). The EC50 value showed that from LDS to DS, the total triterpenoid scavenging capacity of canes and leaves showed a trend of first increasing and then decreasing (Table S6). The total triterpenoids of canes and leaves scavenging capacity for DPPH were as follows: VC > cane at GFS > cane at FS > leaf at GFS > VE > leaf at FS > leaf at LDS > cane at LDS > leaf at DS > cane at DS. The radical scavenging capacity for O_2_^−^· was: VC > cane at FS > leaf at GFS > cane at GFS > leaf at FS > cane at LDS > leaf at LDS > cane at DS > VE > leaf at DS, and for ·OH was VC > cane at GFS > leaf at GFS > cane at FS > leaf at FS > VE > cane at LDS > cane at LDS > leaf at DS > cane at DS (Table S6).

When the concentration of total triterpenoids in the canes increased to 10 mg/mL at different stages, the scavenging capacity of DPPH achieved its maximum, but the scavenging capacity of DPPH for total triterpenoids in leaves continued to increase with the increase in concentration. The changing trend in O_2_^−^· clearance of total triterpenoids in the different stages of the canes and leaves was similar, but the changing trend in ·OH clearance was different, especially at the LDS. Moreover, when the concentration of total triterpenoids in the leaves was lower than 10 mg/mL, the scavenging ability of ·OH free radicals was similar to that of VE, and when the concentration was 20 mg/mL, the scavenging ability of the leaves was lower than that of VE in all stages (Figure 4).

The stronger the reduction ability, the stronger the antioxidant activity of the sample. The total triterpenoids in the different parts at different stages all had reducing ability, and there was a positive correlation between the sample concentration and the reducing ability (Figure 4H,I). The reducing power of the total triterpenoids of the canes and leaves was lower than that of VC and stronger than that of VE. Besides the low concentration of FS and DS, the reducing power of the total triterpenoids in the canes was stronger than that in the leaves.

### 2.4. Determination of Antibacterial Activity

**Figure 5:**
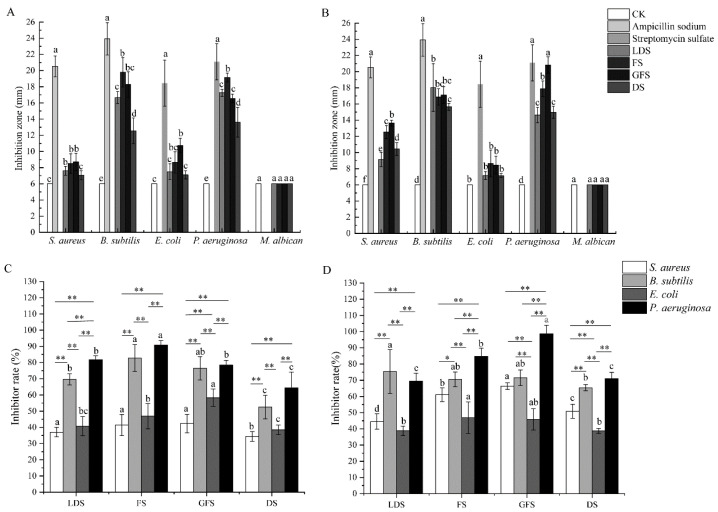
The inhibition zone and inhibitor rate of the total triterpenoid in cane and leaf of *S. sphenanthera* from different stages. (**A**,**C**): cane; (**B**,**D**): leaf. LDS: leaf development stage; FS: flowering stage; GFS: green fruit stage; DS: defoliate stage. Different lowercase letters indicate that the antibacterial effect of total triterpenoid against the same bacteria at different stages is significantly different (*p* < 0.05). ‘*’ (*p* < 0.05) and ‘**’ (*p* < 0.01) indicate the antibacterial effect of total triterpenes on different bacteria during the same stage.

It is well known that the inhibitory capacity is positively correlated with the diameter of the inhibitory zone. The inhibitory zone of the different parts of *S. sphenanthera* was smaller than that of the positive control at different stages, indicating that the inhibitory ability of total triterpenoids was lower than that of antibiotics (Figure 5A,B). All samples showed antibacterial activity against bacteria in vitro, but no antifungal activity (Figure 5A,B). Except for the antibacterial ability of the total triterpenoids in leaves against *Bacillus subtilis*, the diameter of the inhibitory zone of the total triterpenoids from *S. sphenanthera* showed a trend of increasing at first and then decreasing with the development stages, and there were some differences in different stages (Figure 5A,B). At the same time, the tested total triterpenoid extract exhibited strong antibacterial activity with an MIC of 0.31 to 2.50 mg/mL against *Staphylococcus aureus*, *B. subtilis*, *Escherichia coli*, and *Pseudomonas aeruginosa* and MBC values of 1.25 to 15.00 mg/mL (Table S7).

At different stages, the inhibitory abilities of the total triterpenoids in the canes to bacteria were as follows: *P. aeruginosa* > *B. subtilis* > *E. coli* > *S. aureus* (Figure 5C), and in the leaves were as follows: *P. aeruginosa* > *B. subtilis* > *S. aureus* > *E. coli* (Figure 5D). The total triterpenoids in the canes had the highest antibacterial rate against *P. aeruginosa* and *B. subtilis* in FS, which was significantly higher than that in the DS (*p* < 0.05), and the antibacterial rate against *E. coli* in the GFS was higher than that in other stages, and the difference was very significant (*p* < 0.05) (Figure 5A,C). The total triterpenoids in leaves had the highest antibacterial rate against *P. aeruginosa* and *B. subtilis* in GFS, and the highest antibacterial rate against *B. subtilis* in LDS, and were significantly higher than other stages (*p* < 0.05) (Figure 5B,D).

**Figure 6:**
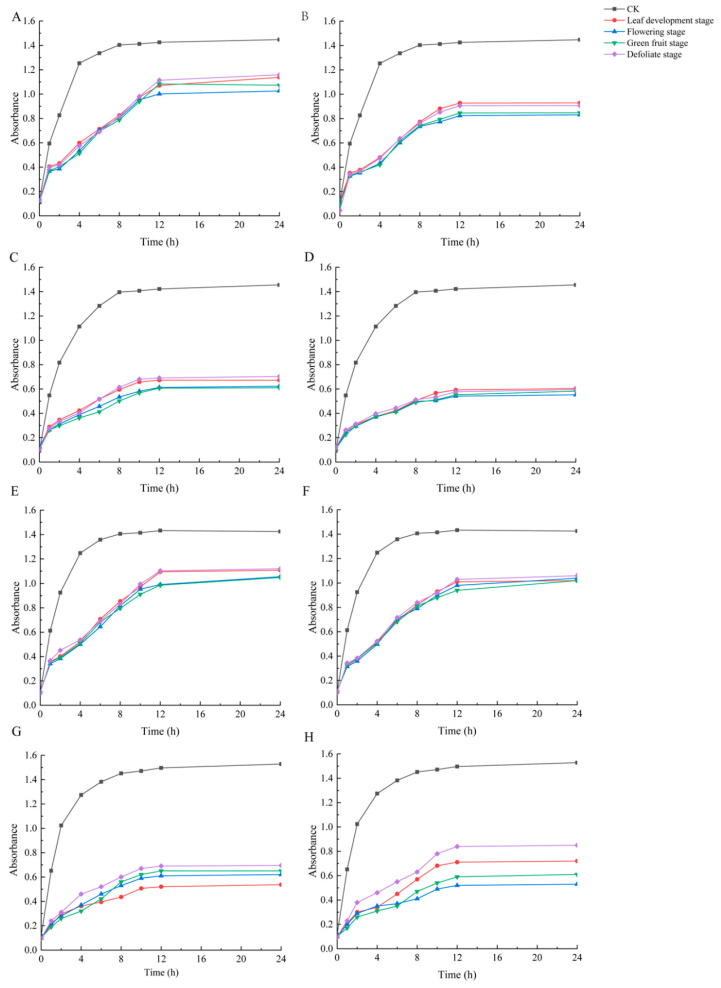
The antibacterial curve of total triterpenoids from the canes and leaves of *S. sphenanthera*. (**A**,**C**,**E**,**G**) canes; (**B**,**D**,**F**,**H**) leaves. (**A**,**B**) *S. aureus*; (**C**,**D**) *B. subtilis*; (**E**,**F**) *E. coli*; (**G**,**H**) *P. aeruginosa*.

The four bacterial species grew well in the blank medium. Compared with the control, there were significant changes after 1 h of intervention with the total triterpenoids (Figure 6). At the beginning of the intervention, the growth of the bacteria was inhibited and the proliferation rate slowed down, but after 4 h, the growth vitality of the bacteria recovered and the bacterial concentration increased significantly (*p* < 0.05). The bacterial concentration entered a dynamic equilibrium after 12 h (Figure 6). In the canes, the growth inhibition curve of the total triterpenoids in the DS was higher than in the other three stages, indicating that the inhibitory effect from the canes in the DS was lower than in other stages; the inhibitory effect of total triterpenoids on the tested bacteria was stronger in the GFS and FS than in the LDS and DS; only the inhibitory effect of the total triterpenoids of the leaves on *P. aeruginosa* in the LDS was stronger than that in other stages (Figure 6A,C,E,G). The total leaves’ triterpenoids at different stages showed that they were generally stronger in the GFS and FS than in the LDS and DS (Figure 6B,D,F,H).

## 3. Discussion

Up to now, a large number of terpenoids with diverse structures and living properties have been identified from the Schisandraceae family [18,19,20,32]. Because its chemical structure is rarely found in the plant kingdom, schinortriterpenoids have become a unique class of metabolites, and more and more monomer components have been detected [33]. Moreover, schisanlactone H has obvious inhibitory activity on HIV and tumor cells and is expected to become the next generation of antitumor drugs [13,34]. However, as a TCM, *S. sphenanthera* is rarely extracted and separated into its single components for use. At present, there are few studies on the extraction and purification of total triterpenoids from *S. sphenanthera*. Triterpenoids in natural plants are insoluble in water, but soluble in organic solvents such as methanol and ethanol. In this study, the solid–liquid ratio, methanol concentration, and extraction time were selected to optimize the ultrasonic extraction process, and the total triterpenoids from the canes and leaves of *S. sphenanthera* were extracted at different growth stages, and the antioxidant and antibacterial activities of the purified total triterpenoids were determined.

The content of total triterpenoids was higher in the leaves than in the stems at different stages, which was consistent with *Allium ursinum* and *Lactuca indica* [35,36]. As the main organ for the transportation of nutrients, the canes provide a pathway for the synthesis and transportation of total triterpenoids, so the content of total triterpenoids is low. However, as the main organ for the accumulation of active compounds, the accumulation of total triterpenoids in the leaves is relatively high. In contrast, the leaves can be used to extract the total triterpenoids, which also helps us to reuse the cane and leaf parts. Moreover, the total triterpenoid content in the canes and leaves of *S. sphenanthera* first decreased, then increased, and then decreased again in different growth stages, especially in the GFS, when the content was higher, which could reduce the damage to the stems and leaves during fruit picking.

Reactive oxygen species (ROS) are important signaling molecules in plants, and the production of ROS will cause cell damage and interfere with important biomolecules, thus affecting normal cell functions [37]. In recent years, antioxidant compounds have been widely used in nutraceutical and pharmaceutical fields because of their resistance to free radical damage. Therefore, the antioxidant properties of the active ingredients of TCM provide an insight into their potential [36,38,39]. Among them, triterpenoids have been shown to have significant antioxidant properties [40], and the free radical scavenging capacity of DPPH, O_2_^−^·, and ·OH radicals are commonly used to assess the antioxidant capacity of medicinal plants [36,38,41]. The antioxidant capacity of triterpenoids is mainly related to the number and position of the hydroxyl groups on the carbon chain; C-9 and C-14 form a seven-membered carbocyclic core and have structures such as lactones [39,42,43]. In this study, the antioxidant capacity of the total triterpenoids in leaves was stronger than that in the canes, which may be due to the different types and contents of triterpenoid monomers, leading to differences in antioxidant capacity [35,36]. Studies have shown that the total triterpenoids of *S. chinensis* had a good therapeutic effect on oxidative cell damage induced by hydrogen peroxide, but their antioxidant capacity was weaker than that of lignans and stronger than that of polysaccharides [44,45]. At present, there is no research on the antioxidant capacity of the total triterpenoids of *S. sphenanthera*, and the results of this experiment further prove the total triterpenoids of *S. sphenanthera* have strong antioxidant capacity.

The indiscriminate use of antibiotics has led to an increase in bacterial resistance, gradually becoming a serious health problem, which adversely affects the human microbiome. As a result, more and more natural products are being used as beneficial alternatives for controlling microbial resistance [46,47]. At present, there are many reports on the antibacterial activity of triterpenoids [41,47,48], but there are few studies on the antibacterial aspects of the active components of *S. sphenanthera*. In this study, the antibacterial activities of the canes and leaves of *S. sphenanthera* against *B. subtilis*, *S. aureus*, *E. coli*, *P. aeruginosa*, and *M. albicans* were measured in vitro. The results showed that the total triterpenoids of *S. sphenanthera* had an inhibitory effect on bacteria, and the antibacterial activity of the leaves’ total triterpenoids was stronger than that of the canes, which was consistent with the contents of total triterpenoids in the canes and leaves as determined by us.

The bactericidal degree of the total triterpenoids of *S. sphenanthera* was judged according to the ratio of MBC/MIC. If the ratio is less than or equal to four, the antibacterial substances are generally considered to be bactericidal. In contrast, the antibacterial substances have antibacterial effects [41]. The total triterpenoids in the canes and leaves had bactericidal effects on *B. subtilis* and *P. aeruginosa*, and antibacterial effects on *S. aureus* and *E. coli* (Table S7). That is, the total triterpenoids in the canes and leaves of *S. sphenanthera* had strong activity against *B. subtilis* and *P. aeruginosa*, but lower inhibitory activity against *S. aureus* and *E. coli*. The difference in antibacterial activity may be caused by the difference in the type and content of total triterpenoids in the various parts at different stages. The total triterpenoids of *S. sphenanthera* in the FS and GFS had the best antibacterial effect on *S. aureus* (Figure 6C,D). In addition to *B. subtilis*, which can be used as probiotics [49], the other three bacteria are pathogenic [50,51,52,53]. While symbiotic *E. coli* is a normal part of the gut microbiota of a healthy host, *E. coli* O157 is both zoonotic and pathogenic [50]. *S. aureus* is a widely used and widespread harmful pathogen, which can cause a variety of invasive diseases such as endometritis and osteomyelitis, and is also considered to be the main pathogen of acute lung injury [51,52]. In addition, the co-infections of *S. aureus* and *P. aeruginosa* are very common in patients with chronic wounds, and their derived extracellular vesicles can promote the pathogenicity of *P. aeruginosa* [53]. To sum up, the total triterpenoids of *S. sphenanthera* may be beneficial natural products in the treatment of invasive human and animal diseases induced by pathogens.

**Figure 7:**
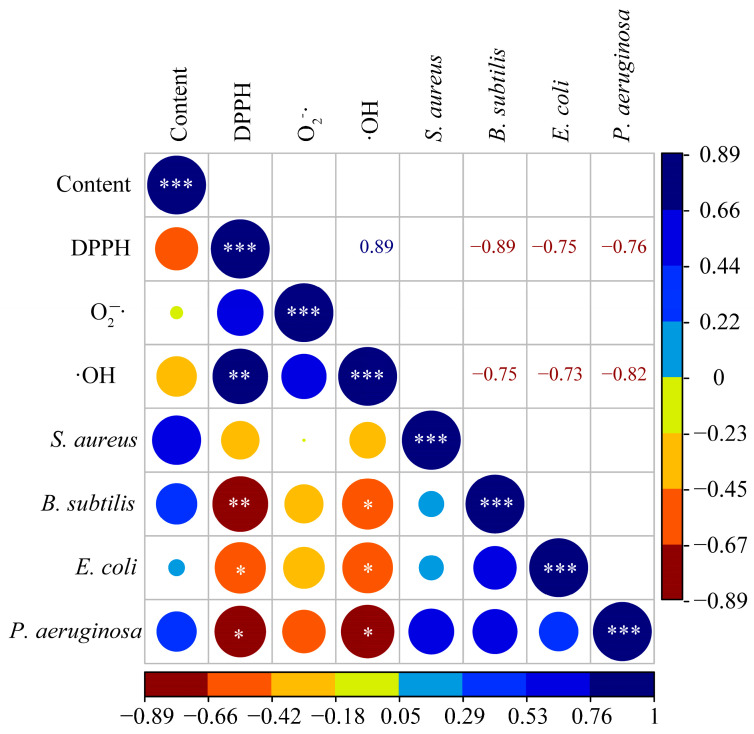
Correlation analysis of total triterpenoid content and antioxidant and antibacterial activity of *S. sphenanthera*. The larger the circle, the stronger the correlation. Those with significant correlations are displayed in the upper right square. “***” indicates extremely significant difference (*p* < 0.001); “**” indicates significant difference (*p* < 0.01); “*” means difference (*p* < 0.05).

If excessive ROS are produced during bacterial metabolism, it will lead to lipid peroxidation of the cell membrane, changes in the cation permeability, and biofilm structure, and damage to bacterial DNA, RNA, proteins, and related cofactors [54]. ROS affects the normal reproduction of bacteria, antioxidant active ingredients can scavenge free radicals and maintain the normal reproduction of bacteria [55]. In this study, a correlation analysis showed that the ability of total triterpenoids to scavenge DPPH free radicals was significantly negatively correlated with the ability to inhibit *B. subtilis* (*p* < 0.01), *E. coli*, and *P. aeruginosa* (*p* < 0.05). and the ability to scavenge ·OH free radicals was also negatively correlated with the ability to inhibit these three bacteria (*p* < 0.05) (Figure 7). These results indicated that the antioxidant capacity of the total triterpenoids of *S. sphenanthera* could inhibit bacterial reproduction to a certain extent. However, we did not find a significant correlation between total triterpenoid content and bioactivity, which may be because we used purified total triterpenoids for later bioactivity studies. Based on the results of the antibacterial and antioxidant experiments, the related triterpenoid components can be identified in the follow-up, and the related mechanism of the antioxidative and antibacterial activities of the canes and leaves of *S. sphenanthera* can be further explored.

## 4. Materials and Methods

### 4.1. Plant Materials

The canes and leaves of *S. sphenanthera* were picked from the plantation at Shaanxi Normal University. The *S. sphenanthera* plants were transplanted from the Qinling Mountains and had been grown for 15 years. The canes and leaves of *S. sphenanthera* were picked in the leaf development stage (LDS) (15 April 2023), flowering stage (FS) (2 May 2023), green fruit stage (GFS) (17 June 2023), and defoliate stage (DS) (19 November 2023). The materials were dried and stored at −80 °C for later use.

### 4.2. Extraction of the Total Triterpenoids

#### 4.2.1. Extraction Method for Triterpenoid Optimization

Three factors were selected in this study for single-factor experiments, including the solid–liquid ratio (1:5 g/mL, 1:10 g/mL, 1:20 g/mL, 1:30 g/mL, 1:40 g/mL, and 1:50 g/mL), methanol concentration in solvent (50%, 60%, 70%, 80%, 90%, and 100%), and extraction time (10 min, 20 min, 30 min, 40 min, 50 min, 60 min, 70 min, 80 min, and 90 min). The ultrasonic temperature was 60 °C, and the optimal extraction process was determined by the response surface method (RSM) [29], which provided the basis for subsequent extraction. After adding methanol solvent to the sample according to the ratio of material to liquid, it was necessary to stir evenly with a glass rod. The 0.5 g sample was used to extract the total triterpenoids from the canes and leaves of *S. sphenanthera* by using the optimal method obtained by RSM to obtain the crude extract of total triterpenoids.

#### 4.2.2. Determination of Total Triterpenoid Content

Oleanolic acid was used as a standard to draw a standard curve [36]. The total triterpenoid extraction solution (0.2 mL) was measured and transferred to test tubes. Then, 0.2 mL 5% vanillin-glacial acetic acid solution, and 0.8 mL perchloric acid were added and mixed. The resulting solution was heated in 60 °C water for 15 min and then cooled to room temperature, followed by addition of glacial acetic acid to 5 mL. Absolute ethanol was the control. The absorbance of the triterpenoid extraction solutions was measured at 560 nm and the extraction rate was calculated by Equation (3).

(3) $$Y=\left[\left(M\times 5\times n\right)÷\left(m\times 1000\right)\right]\times 100$$

where *Y* is the extraction rate of total triterpenoid, *M* is the total triterpenoid weight on the standard curve corresponding to the absorbance of the sample (mg), *n* is the dilution ratio, and *m* is the sample weight (g).

#### 4.2.3. Purification of Total Triterpenoid Extract

The D-101 type macroporous resin was used to purify the crude extracts of total triterpenoids extracted from the canes and leaves of *S. sphenanthera* [29].

### 4.3. Antioxidant Ability

#### 4.3.1. The Scavenging Ability of Three Radicals

The improved method in our laboratory was used to detect the scavenging ability of the purified total triterpenoid extract on DPPH radical, hydroxyl radical, and superoxide anion radical [8]. The purified total triterpenoids were diluted with distilled water to different concentrations (2 μg/mL, 4 μg/mL, 6 μg/mL, 8 μg/mL, 10 μg/mL, 20 μg/mL, 40 μg/mL, 60 μg/mL, 80 μg/mL, and 100 μg/mL). The antioxidant activities of total triterpenoids were compared with vitamin C (VC) and vitamin E (VE) as controls, and the ability to scavenge free radicals was calculated by Equation (4).

(4) $$\mathrm{Clearance}\;\left(\%\right)=\left[A_{0}-\left(A_{i}-A_{j}\right)\right]/A_{0}\times 100$$

where *A* is the clearance, *A*_0_ is the absorbance value of the blank solution, *A_i_* is the absorbance of the purified total triterpenoids solution and the free radicals at the same time, and *A_j_* is the absorbance of the total triterpenoids without the free radicals. All experiments were repeated three times and the EC_50_ value was calculated.

#### 4.3.2. Determination of Reduction Power

The improved method in our laboratory was used to detect the reduction ability of total triterpenoids after purification [8]. A measure of 1 mL total triterpenoid solution was pipetted and mixed with 2.5 mL potassium ferricyanide (1%) and 2.5 mL phosphate buffer. The mixture was heated in 50 °C water for 20 min and then cooled to room temperature, followed by the addition of 2.5 mL trichloroacetic acid solution (10%). The resulting solution was centrifuged at 3000 rpm for 10 min. The supernatant (100 μL) was measured and mixed with 20 μL anhydrous iron(III) chloride (FeCl_3_) (0.1%), and 80 μL distilled water for 10 min. The absorbance was measured at 700 nm.

### 4.4. Antibacterial Ability

#### 4.4.1. Strain Activation and Bacterial Suspension Preparation

In this study, 5 strains were selected for the evaluation of antibacterial activity, including 4 bacteria species (*Staphylococcus aureus*, *Bacillus subtilis*, *Escherichia coli*, and *Pseudomonas aeruginosa*), and a fungus (*Monilia albicans*). Strain activation: the colonies were picked under sterile conditions and inoculated into solid and liquid media. The bacteria were cultured at 37 °C incubator (Jiangnan Instrument Factory, Ningbo, China) for 24 h, and the fungi were cultured at 28 °C for 48 h. Preparation of bacterial suspension: the activated bacterial solution (1 mL) was diluted with 0.9% normal saline.

#### 4.4.2. Determination of Inhibition Zone

Inhibition zones of total triterpenoids were detected using an agar-well diffusion assay [56]. A certain number of sterilized filter paper pieces were each soaked in sterilized water, total triterpene solution (20 mg/mL), and antibiotic solution (10 mg/mL) for 10 min. Then the soaked filter paper was placed on a medium coated with bacterial solution (100 μL) under the same culture conditions as the activation conditions of the strain, and the diameter of the inhibition band was determined. Water was a blank control, and ampicillin sodium and streptomycin sulfate were selected as positive controls for Gram-positive bacteria and Gram-negative bacteria, respectively. If the diameter of the bacteriostatic zone is greater than 6 mm, it indicates that the sample has certain bacteriostatic ability. The bacteriostatic rate was calculated according to Equation (5).

(5) $$\mathrm{Inhibition}\;\mathrm{rate}\;\left(\%\right)=B_{0}/B_{1}\times 100\%$$

where *B* is the inhibition rate, *B*_0_ is the diameter of the inhibition zone of the experimental group (mm), and *B*_1_ is the diameter of the inhibition zone of positive control (mm).

#### 4.4.3. Determination of Minimum Inhibitory Concentration (MIC) and Minimum Bactericidal Concentration (MBC)

The minimum inhibitory concentration (MIC) and minimum bactericidal concentration (MBC) of total triterpenoids were determined by standard microdilution, and total triterpenoid solutions were diluted to different concentrations (10.00 mg/mL, 5.00 mg/mL, 2.50 mg/mL, 1.25 mg/mL, 0.63 mg/mL, 0.31 mg/mL, 0.16 mg/mL, 0.08 mg/mL, and 0.04 mg/mL); they were added to the 96-well plate together with the liquid medium containing bacteria, and the final total volume of each well was 100 μL. The plate was incubated in a 37 °C incubator until no colonies grew. Water was the blank control, and ampicillin sodium and streptomycin sulfate were selected as a positive control for Gram-positive bacteria and Gram-negative bacteria, respectively. The lowest concentration of total triterpenoids in each sample showing no growth was taken as its MIC. The obtained bacteria–sample mixture with MIC or above the concentration of total triterpenoids (100 μL) was coated on a solid medium and cultured in a 37 °C incubator for 24 h. The lowest concentration without growth after subculture was its MBC, which indicated that the killing rate of the original inoculum was >99.9%.

### 4.5. Statistical Analysis

All experiments were performed in triplicate, and the results were expressed as mean ± standard deviation (SD). In this study, Design-Expert.V8.0.6.1 software was used for RSM, SPSS 19 software for single-factor analysis of variance and multiple comparisons, R 4.3.1 software was used to analyze the correlation between extraction rate, antioxidant activity, and antibacterial activity, and Origin 2018 and GraphPad Prism 8 software for graphing.

## 5. Conclusions

This study is the first attempt to optimize the extraction process of total triterpenoids from the canes and leaves of *S. sphenanthera* and investigate their antioxidant and antibacterial activities. The RSM-optimized extraction method for total triterpenoids, using an ultrasonic methanol solution, formed the basis for extracting triterpenoids from the canes and leaves of *S. sphenanthera*. The growth and developmental stage of *S. sphenanthera* affects the total triterpenoid content in the canes and leaves and the antioxidant and antibacterial activities. These data provide a good indication for determining the best harvest time for the different parts. The total triterpenoid content in the leaves was higher than that in the canes, and the GFS was the best harvest time. Our study also showed that the total triterpenoids in the canes and leaves of *S. sphenanthera* had strong antioxidant and antibacterial effects during FS and GFS. This study may provide a theoretical basis and data support for the future development and utilization of *S. sphenanthera* canes and leaves.

## Data Availability

The data presented in this study are available in article and Supplementary Materials.

## Supplementary Materials

The following supporting information can be downloaded at: https://www.mdpi.com/article/10.3390/molecules29102199/s1, Table S1: The optimization design and results of response surface analysis experiments of ultrasound-assisted triterpenoid extraction from the canes of *S. sphenanthera*; Table S2: The optimization design and results of response surface analysis experiments of ultrasound-assisted triterpenoid extraction from the leaves of *S. sphenanthera*; Table S3: Variance analysis table of the regression equation for the canes of *S. sphenanthera*; Table S4: Variance analysis table of the regression equation for the leaves of *S. sphenanthera*; Table S5: Purification effect of triterpenoids from *S. sphenanthera* by D-101 macroporous resin; Table S6: Antioxidation of three free radicals by total triterpenoids from canes and leaves of *S. sphenanthera*; Table S7: The MIC and MBC results of total triterpenes of *S. sphenanthera* against four bacteria.
