# Supplementary material for: Optimization of the Extraction Process and Biological Activities of Triterpenoids of Schisandra sphenanthera from Different Medicinal Parts and Growth Stages
Source: Molecules. 2024 May 8;29(10):2199. doi: 10.3390/molecules29102199 (PMC11123978; doi:10.3390/molecules29102199)
Supplement: Supplementary file 1 [file molecules-29-02199-s001.zip › molecules-2975997-supplementary.pdf]

Table S1. The optimization design and result of response surface analysis experiments of ultrasound-assisted triterpenoid extraction from the cane of *S. sphenanthera*.

Number	A (g/mL) Solid-liquid ratio	B (%) Methanol concentration	C (min) Extraction time	(%) Extraction rate
1	20.00	85.00	40.00	0.77
2	40.00	85.00	40.00	0.50
3	30.00	80.00	50.00	1.09
4	30.00	80.00	50.00	1.09
5	30.00	80.00	50.00	1.12
6	30.00	80.00	50.00	1.13
7	30.00	80.00	50.00	1.14
8	13.18	80.00	50.00	0.70
9	20.00	75.00	60.00	0.75
10	30.00	80.00	66.82	0.96
11	30.00	80.00	50.00	1.14
12	40.00	85.00	60.00	0.76
13	30.00	71.59	50.00	0.96
14	46.82	80.00	50.00	0.54
15	40.00	75.00	40.00	0.75
16	30.00	80.00	33.18	0.72
17	40.00	75.00	60.00	0.92
18	20.00	85.00	60.00	0.74
19	20.00	75.00	40.00	0.93
20	30.00	88.41	50.00	0.77

Table S2. The optimization design and result of response surface analysis experiments of ultrasound-assisted triterpenoid extraction from the leaf of *S. sphenanthera*.

Number	A (g/mL) Solid-liquid ratio	B (%) Methanol concentration	C (min) Extraction time	Extraction rate
1	30.00	95.00	80.00	1.34
2	20.00	90.00	70.00	1.62
3	36.82	90.00	70.00	1.12
4	10.00	85.00	80.00	0.97
5	20.00	81.59	70.00	1.05
6	20.00	90.00	53.18	0.28
7	20.00	98.41	70.00	1.30
8	10.00	95.00	80.00	1.36
9	20.00	90.00	70.00	1.59
10	3.18	90.00	70.00	0.84
11	20.00	90.00	70.00	1.65
12	30.00	85.00	80.00	1.36
13	20.00	90.00	86.82	1.32
14	10.00	85.00	60.00	0.36
15	20.00	90.00	70.00	1.62
16	20.00	90.00	70.00	1.66
17	10.00	95.00	60.00	0.68
18	30.00	85.00	60.00	0.53
19	20.00	90.00	70.00	1.68
20	30.00	95.00	60.00	0.62

Table S3. Variance analysis table of the regression equation for the cane of *S. sphenanthera*.

Source	Sum of square	Degree of freedom	Mean	*F* value	*p-value*	Significance
Model	0.744	9	0.083	69.945	< 0.0001	**
A_1- Solid-liquid ratio	0.020	1	0.020	16.885	0.002	**
B_1- Methanol concentration	0.058	1	0.058	49.427	< 0.0001	**
C_1- Extraction time	0.028	1	0.028	23.557	0.001	**
A_1B_1	0.007	1	0.007	6.097	0.033	*
A_1C_1	0.053	1	0.053	45.017	< 0.0001	**
B_1C_1	0.007	1	0.007	5.715	0.038	*
A_1^2	0.426	1	0.426	360.122	< 0.0001	**
B_1^2	0.104	1	0.103	87.549	< 0.0001	**
C_1^2	0.134	1	0.133	112.957	< 0.0001	**
Residual	0.012	10	0.001			
Lack of fit	0.009	5	0.002	3.487	0.098	-
Pure error	0.003	5	0.001			
Cor Total	0.756	19				

"**" means extremely significant ($p < 0.01$); "*" means significant ($p < 0.05$); "-" means not significant

Table S4. Variance analysis table of the regression equation for the leaf of *S. sphenanthera*.

Source	Sum of square	Degree of freedom	Mean	*F* value	*p-value*	Significance
Model	3.987	9	0.443	174.299	< 0.0001	**
A_2- Solid-liquid ratio	0.064	1	0.064	25.302	0.0005	**
B_2- Methanol concentration	0.108	1	0.108	42.666	< 0.0001	**
C_2-Extraction time	1.539	1	1.539	605.516	< 0.0001	**
A_2B_2	0.051	1	0.051	20.235	0.001	**
A_2C_2	0.009	1	0.009	3.488	0.091	-
B_2C_2	0.0002	1	0.0002	0.073	0.792	-
A_2^2	0.836	1	0.836	329.070	< 0.0001	**
B_2^2	0.426	1	0.426	167.687	< 0.0001	**
C_2^2	1.349	1	1.349	530.868	< 0.0001	**
Residual	0.025	10	0.003			
Lack of fit	0.021	5	0.004	4.234	0.070	-
Pure error	0.005	5	0.001			
Cor Total	4.013	19				

"**" means extremely significant ($p < 0.01$); "-" means not significant

Table S5. Purification effect of triterpenoids from *S. sphenanthera* by D-101 macroporous resin.

Sample	Triterpenoid before purification (mg)	Triterpenoid after purification (mg)	Purification rate (%)	Purity of product before purification (%)	Product purity (%)
Cane of LDS	50.00	11.38	22.76	24.96	72.14
Cane of FS	50.00	16.62	33.24	26.59	77.63
Cane of GFS	50.00	11.85	23.70	25.65	79.52
Cane of DS	50.00	12.58	25.16	28.12	70.87
Leaf of LDS	50.00	15.84	31.74	20.97	73.39
Leaf of FS	50.00	13.72	27.43	27.93	80.44
Leaf of GFS	50.00	12.09	24.17	28.48	76.61
Leaf of DS	50.00	11.70	23.39	27.82	69.55

LDS: leaf development stage; FS: flowering stage; GFS: green fruit stage; DS: defoliate stage.

Table S6. Antioxidation of three free radicals by total triterpenoids from canes and leaves of *S. sphenanthera*.

	Sample	Regression equation	R^2	EC_{50}(mg/mL)
DPPH	VC	$y = 0.00001x^3-0.003x^2+0.526x+66.536$	0.994	$1.14×10^{-3}$ g
	VE	$y = -0.007x^3+0.043x^2+5.926x+11.644$	0.989	4.97 f
	Cane of LDS	$y = -0.046x^3+1.195x^2-3.061x+6.376$	0.945	20.39 c
	Cane of FS	$y = 0.021x^3-0.8x^2+10.855x+15.123$	0.996	3.99 fg
	Cane of GFS	$y = 0.013x^3-0.703x^2+12.369x+12.048$	0.992	2.85 fg
	Cane of DS	$y = -0.024x^3+0.573x^2+0.174x+10.165$	0.968	39.43 a
	Leaf of LDS	$y = 0.006x^3-0.313x^2+6.187x+13.4$	0.941	15.32 d
	Leaf of FS	$y = 0.004x^3-0.26x^2+5.843x+16.745$	0.984	12.22 e
	Leaf of GFS	$y = 0.002x^3-0.343x^2+10.154x+7.402$	0.983	4.83 f
	Leaf of DS	$y = 0.11x^3-0.391x^2+5.774x+10.551$	0.986	28.90 b
O_2^-·	VC	$y = -7.314x^3-0.001x^2+0.557x+10.587$	0.993	0.11 f
	VE	$y = 0.007x^3-0.251x^2+4.395x+13.044$	0.944	29.40 b
	Cane of LDS	$y = -0.002x^3-0.075x^2+4.499x+19.170$	0.998	11.11 d
	Cane of FS	$y = 0.009x^3-0.397x^2+6.556x+22.939$	0.979	5.72 e
	Cane of GFS	$y = 0.018x^3-0.717x^2+9.845x+10.665$	0.988	7.92 de
	Cane of DS	$y = -0.001x^3-0.186x^2+6.296x+3.799$	0.993	16.08 c
	Leaf of LDS	$y = 0.022x^3-0.849x^2+10.293x+5.044$	0.971	13.14 cd
	Leaf of FS	$y = 0.006x^3-0.346x^2+7.417x+4.691$	0.992	11.06 d
	Leaf of GFS	$y = 0.007x^3-0.298x^2+5.081x+27.130$	0.974	5.95 de
	Leaf of DS	$y = 0.010x^3-0.350x^2+4.184x+25.312$	0.871	44.73 a
·OH	VC	$y = 0.00009x^3-0.017x^2+1.699x+12.960$	0.972	$1.96×10^{-2}$ cd
	VE	$y = 0.006x^3-0.311x^2+7.927x+12.450$	0.929	4.28 c
	Cane of LDS	$y = 0.019x^3-0.658x^2+9.055x+16.760$	0.983	4.34 c
	Cane of FS	$y = -0.003x^3+0.005x^2+4.243x+23.460$	0.974	4.11 c
	Cane of GFS	$y = 0.21x^3-0.885x^2+12.183x+19.927$	0.996	2.11 c
	Cane of DS	$y = 0.012x^3-0.511x^2+6.868x+17.524$	0.922	18.52 a
	Leaf of LDS	$y = 0.023x^3-0.864x^2+10.360x+8.559$	0.967	11.90 b
	Leaf of FS	$y = 0.005x^3-0.400x^2+9.147x+12.984$	0.940	4.26 c
	Leaf of GFS	$y = -0.002x^3-0.033x^2+4.169x+27.506$	0.963	3.41 c
	Leaf of DS	$y = 0.024x^3-0.877x^2+9.937x+8.829$	0.900	13.34 b

The same lowercase letters indicate that the clearance rate of a certain free radical in different parts of different periods is not significantly different, while the different lowercase letters indicate significant differences ($p < 0.05$). LDS: leaf development stage; FS: flowering stage; GFS: green fruit stage; DS: defoliate stage.

Table S7. The MIC and MBC results of total triterpenes of *S. sphenanthera* against four bacteria.

Sample	*S. aureus* (mg/mL)			*B. subtilis* (mg/mL)			*E. coli* (mg/mL)			*P. aeruginosa* (mg/mL)		
	MIC	MBC	MBC/MIC	MIC	MBC	MBC/MIC	MIC	MBC	MBC/MIC	MIC	MBC	MBC/MIC
Cane of LDS	2.50	15.00	6	1.25	5.00	4	2.50	15.00	6	0.63	2.50	4
Cane of FS	2.50	10.00	4	0.63	2.50	4	2.50	15.00	6	0.31	1.25	4
Cane of GFS	2.50	10.00	4	0.63	2.50	4	1.25	10.00	8	0.63	2.50	4
Cane of DS	2.50	15.00	6	1.25	10.00	8	2.50	15.00	6	1.25	5.00	4
Leaf of LDS	2.50	15.00	6	0.63	2.50	4	2.50	15.00	6	1.25	5.00	4
Leaf of FS	1.25	5.00	4	0.63	2.50	4	2.50	15.00	6	0.63	2.50	4
Leaf of GFS	1.25	5.00	4	0.63	2.50	4	2.50	10.00	4	0.31	1.25	4
Leaf of DS	1.25	10.00	8	1.25	2.50	2	2.50	15.00	6	1.25	5.00	4
